# Antibacterial Activity of In Situ Prepared Chitosan/Silver Nanoparticles Solution Against Methicillin-Resistant Strains of *Staphylococcus aureus*

**DOI:** 10.1186/s11671-018-2482-9

**Published:** 2018-03-02

**Authors:** Viktoriia Holubnycha, Oksana Kalinkevich, Olena Ivashchenko, Maksym Pogorielov

**Affiliations:** 1 Sumy State University, Medical Institute, 2, R-Korsakova street, Sumy, 40007 Ukraine; 2 Institute of Applied Physics, National Academy of Sciences of Ukraine, 58, Petropavlivska St., Sumy, 40000 Ukraine; 3 NanoBioMedical Centre, Adam Mickiewicz University, Umultowska 85, 61-614 Poznań, Poland

**Keywords:** Chitosan, Silver nanoparticles, CTAB, MRSA, Antibacterial activity

## Abstract

**Background:**

Investigation of new effective drugs against the methicillin-resistant strains of *Staphylococcus* aureus (MRSA) is an urgent issue of modern medicine. Antiseptics as an alternative of antibiotics are strong, sustained, and active preparations against resistant strains and do not violate microbiocenosis.

**Materials and Methods:**

The activity of in situ prepared chitosan-Ag nanoparticles (Ag NPs) solution with different component ratio was tested against MRSA isolated from patients. Ag NPs were synthesized via chemical reduction method using green chemistry approach. In order to improve antimicrobial activity and dispersibility of Ag NPs, surface modification of Ag NPs by cetrimonium bromide (CTAB) was performed.

Ag NPs and chitosan-Ag NPs solution were characterized using X-ray diffraction, transmission electron microscopy, infrared spectroscopy, and spectrophotometric measurements.

**Results and Conclusions:**

The results of XRD, FTIR, UV–Vis, and TEM measurements confirmed the chemical composition of chitosan and Ag NPs and their high purity.

Chitosan-AgNPs solutions have shown their superior antimicrobial efficacy compared to its pure forms. At the same time, in situ preparation of chitosan-Ag NPs solution (chitosan powder 6.0 μg/ml, Ag/CTAB NPs) was not possible due to the precipitation of the components. This result is very promising and may be considered as an effective solution in fighting against drug-resistant bacteria.

## Background

Infections remain a major cause of morbidity and mortality worldwide despite the presence of a significant number of antibiotics and antiseptics. In moderate and severe infections, antibiotic therapy is usually initiated empirically before obtaining results of bacteriological examination. Constant use of antibiotics created favorable conditions for the selection and multiplication of antibiotic-resistant microorganisms [22]. High prevalence of multidrug resistance to agents of all infectious processes is documented nowadays [6]. The most notorious multidrug-resistant bacterium is methicillin-resistant *Staphylococcus aureus* (MRSA) [9]. The pathogen is responsible for a broad spectrum of human and animal diseases ranging from the skin infections to such severe disorders as pneumonia, endocarditis, and septicemia, and these infections may impact human health [32]. Analysis of etiological causes of infections in patients with inadequate therapy revealed that therapy was inadequate in 32.6% cases of MRSA-based infections [12] and associated with 3–4 billion US dollars in annual health care costs [32].

Investigation of new effective drugs against the MRSA is an urgent issue of modern medicine. Antiseptics as an alternative of antibiotics are strong, sustained, and active preparations against resistant strains and do not violate microbiocenosis. Overcoming these problems requires new and innovative preparations. The approach of combining different mechanisms of antibacterial action by designing hybrid nanomaterials provides a new paradigm in the fight against resistant bacteria [18]. Metals, such as copper and silver, are extremely toxic to bacteria at exceptionally low concentrations. Due to biocidal activity, metals have been widely used as antimicrobial agents in a multitude of applications related to agriculture, healthcare, and the industry in general. Unlike other antimicrobial agents, metals are stable under conditions currently found in the industry allowing their use as additives [19].

The antimicrobial properties of silver have been known from antiquity, and increasing antibiotic resistance of bacteria and the ineffectiveness of synthetic antibiotics against some bacterial strains have led to the reemergence of interest in silver, silver salts, silver compounds, and nanocrystalline silver as antibacterial agents. Silver nanoparticles (Ag NPs) have significant antibacterial and antifungal effect [26]. Ag NPs show synergism with other antibiotics and antiseptics (ceftazidime, streptomycin, kanamycin, polymyxin) [25, 38]. But J. Jains showed that chloramphenicol decreases antibacterial effect of Ag NPs solution [16].

The main disadvantages that limit the use of Ag NPs are their easy aggregation, the uncontrolled release of silver ions, and their cytotoxicity potential [40]. Combination of Ag NPs with natural agents, such as chitosan, propolis, clays, or zeolites [33, 35], provides additional effects. The combination of polymers and nanosilver may synergistically improve their antimicrobial effects, and the use of in situ synthesis methods allows its incorporation into the polymer matrix attaining uniform distributions and avoiding aggregation [28].

In recent years, the efficiency of green chemistry methods for synthesis of metallic NPs has increased significantly [1]. Plant extracts are often used as reducing, stabilizing, and capping agents [23] providing cost-effective and environmentally benign methods for NPs synthesis. Among plant extracts, ginger extract is of great scientific interest thanks to its chemical and biological properties [8]. Leaf extract of ginger has already been used for synthesis of silver NPs [37]; however, the produced particles had a rather wide particle size distribution (10–100 nm). Ginger rhizome is widely used as a spice and a folk medicine; its extract contains specific phenolic compounds: gingerol and its derivatives, a number of bioactive phenolic and non-phenolic constituents [31]. These compounds exhibit a broad spectrum of activities including antimicrobial, antifungal, and antiviral ones. Rhizome ginger extract seems to be a very promising substratum for development of bioactive and biocompatible nanoparticles, because it demonstrates also antioxidant and anti-inflammatory properties.

Chitin and chitosan are promising materials for medical applications due to their bacteriostatic/bactericide properties and biocompatibility with human tissues [20]. Chitosan is a derivative of chitin, which can be obtained by chitin deacetylation. Both of them contain same monomers, *N*-acetyl-2-amino-2-deoxy-D-glucopyranose and 2-amino-2-deoxy-D-glucopyranose, which differ in the proportion of acetylated and deacetylated monomers. Chitosan is a promising material for forming composites with different substances, including metal nanoparticles such as Ag and Cu [33]. On the other hand, сetrimonium bromide (CTAB) can stabilize nanoparticles in solution and decrease the toxicity of some nanoparticles, such as ZnO, TiO2, and Ni [17]. But the data about antibacterial activities of CTAB-NPs complex are limited [7].

The purpose of this research is to find optimal ratio of chitosan and Ag NPs, modified by CTAB for solution composition (chitosan/Ag) that would be active against MRSA clinical strains.

## Methods

### Materials

Silver nitrate, L-ascorbic acid, and cetrimonium bromide (C_16_H_33_)N(CH_3_)_3_Br (CTAB) were purchased from Sigma-Aldrich and used as received. Ginger (*Zingiber officinale*, *Zingiber acae*) rhizome was purchased in a local supermarket (Poznan, Poland). Chitosan 200 kDa, deacetylation degree 82% was purchased from CJSC “Bioprogress” (Russia, Moscow) and used without further purification. Ultrapure water (resistivity > 17 MΩcm^− 1^) from a GZY-P10 water system was used throughout the experiments. All media and disks with antibiotics were purchased from Hi Media (India).

### In Situ Preparation of Chitosan/Ag NPs Solutions

To prepare the chitosan/Ag solutions in situ, Ag NPs were synthesized and modified at first.

#### Synthesis of Ag NPs

Ag NPs were synthesized via chemical reduction method using green chemistry approach. Following this approach, we used ginger (*Zingiber officinale*) extract as a surfactant and ascorbic acid (vitamin C) as a reducing agent. To prepare ginger rhizome extract, 250 g of rhizome was washed thoroughly with distilled water and then cut into small pieces. Chopped ginger rhizome was kept in a water-ethanol solution (250 ml, 1:1 ratio) for 5 days (at room temperature, in dark place). Then, supernatant was vacuum filtered (through a Whatman filter paper) and stored (at 4 °C). To synthesize Ag NPs, silver nitrate (840 mg) was dissolved in water (20 ml) and ginger rhizome extract (20 ml) was added. Then, a mixture of solution of L-ascorbic acid (10%, 10 ml) and ginger extract (20 ml) was drop added to the silver nitrate solution under magnetic steering. The reaction mixture turned dark. Then, it was heated (60 °C, 1.5 h) under reflux. Then, freshly synthesized Ag NPs were washed with water, until pH reached 7, using centrifugation (4000 rpm, 30 min).

In order to improve antimicrobial activity and dispersibility of Ag NPs, surface modification of Ag NPs by CTAB, which is well-known due to its surface-active and antiseptic properties, was performed [17]. Typically, the dispersion of Ag NPs (3 ml, 76.4 mg/ml) was mixed with CTAB solution (20 ml, 6.7 mg/ml) and sonicated (3 h). Then, supernatant was collected for UV–Vis measurements and Ag NPs were washed with water, using centrifugation (4000 rpm, 30 min), three times. Content of CTAB in the supernatant was determined using the spectrophotometric technique (UV–Vis) by monitoring the intensity of the 190-nm peak. Adsorptivity of Ag NPs (in mg/g) towards CTAB was calculated from the difference between the initial CTAB content in the solution and its content in the supernatant after interaction with the sample. The adsorptivity and CTAB loading content were calculated from the following equations:

Adsorptivity (mg/g) = (weight of CTAB in solution − weight of CTAB in supernatant)/(weight of Ag NPs),

CTAB loading content (%) = (1 − (weight of Ag NPs)/(weight of CTAB loaded Ag NPs)) × 100%.

#### In Situ Preparation of Chitosan/Ag NPs Solutions

To obtain chitosan/Ag NPs solutions, 200 kDa chitosan (1 g) was dissolved in 2% acetic acid (100 ml) at room temperature during 24 h to form 1% chitosan solution. Two samples of Ag NPs were used in experiments—pure Ag NPs and Ag NPs-CTAB.

### Physicochemical Characterization of Ag NPs and Chitosan

Powder X-ray diffraction (XRD) studies were conducted on an Empyrean diffractometer (PANalytical), using Cu Kα radiation (1.54 Å), a reflection-transmission spinner (sample stage), and PIXcel 3D detector, operating in the Bragg–Brentano geometry. The 2Theta scans were recorded at room temperature at angles ranging from 10° to 95° with a step size of 0.007°, in continuous scan mode.

Transmission electron microscopy (TEM) measurements were performed using a JEM-ARM-200F transmission electron microscope operating at an accelerating voltage of 200 kV.

The infrared spectra were obtained using a Tensor 27 (Bruker Optics) spectrometer equipped with a global source and MCT detector. Samples were prepared using potassium bromide as a matrix material and were mixed in proportions of 1 mg of sample to 200 mg KBr. Pellets were prepared using the standard technique under a pressure of 10 ton/cm^2^ with a barrel of 16 mm in diameter. The measurements were performed at room temperature. For each spectrum, 512 scans in the spectral range of 4000–400 cm^− 1^ were taken with a resolution of 4 cm^− 1.^ The data were processed using the Opus software package.

Spectrophotometric measurements (UV–Vis) were performed using UV/VIS/NIR Spectrometer Lambda 950 (Perkin Elmer) at wavelengths 200–800 nm with water as referred solution.

### Microbiological Tests

#### Bacterial Culture

Bacterial cultures were collected from the region of the middle nasal meatus and from the throat of the 70 inpatients by use of sterile cotton-tipped wire swabs. The specimens were transported immediately to the laboratory in transport media and then inoculated on blood agar. Bacterial cultures were identified morphologically and biochemically by standard laboratory procedures according to the Manual of Methods for General Bacteriology in the bacteriological laboratory of Sumy State University. We isolated 50 *Staphylococcus aureus* strains. Each culture underwent Gram staining and was tested for production of catalase, free coagulase, yellow pigment, mannitol fermentation, growth on high salt concentration, and lipase production on egg yolk agar medium (Hi Media, Mumbai).

#### Antimicrobial Susceptibility Testing

Antibiotic susceptibility tests were performed on all *S*. *aureus* isolates to determine their antibiotic resistance profiles. The Kirby-Bauer disk diffusion method was used to assess the antibiotic susceptibility of the isolates. Antimicrobial susceptibility testing was carried out on Muller-Hinton agar against azithromycin, levofloxacin, clarithromycin, ciprofloxacin, and methicillin (National Committee for Clinical Laboratory Standards, 1999). Fresh overnight cultures were prepared and used in tests. Standard strain of *S*. *aureus* ATCC 25923 was used as control. An aliquot (100 μL) from each isolate suspension was spread plated on Mueller Hinton agar. Antibiotic discs were gently pressed onto the inoculated Mueller Hinton agar to ensure intimate contact with the surface, and the plates were incubated aerobically at 37 °C for 18–24 h. Inhibition zone diameters were measured. Clinical strains were categorized as susceptible and resistant according to evaluation criteria developed by the Clinical and Laboratory Standards Institute (CLSI) guidelines [24]. The strains of *Staphylococcus aureus* which were found to be resistant to methicillin were screened as MRSA.

#### Determination of Minimum Inhibitory Concentrations of Chitosan-Ag NPs Solutions

Antimicrobial activities of chitosan solution, Ag NPs, and chitosan-Ag NPs solutions were determined according to the recommendations of NCCLS (1999) by the use of a broth macrodilution method. We determined the minimum inhibitory concentration (MIC) for test solutions against each methicillin-resistant *Staphylococcus aureus* (a total of 10 MRSA strains). The tube with the lowest concentration that completely inhibits visual growth of bacteria (no turbidity) was considered as the MIC.

Briefly, at the beginning, seven concentrations of pure Ag NPs and Ag/CTAB NPs were prepared using nutrient broth with the 2-fold serial dilution method. There were three identical rows of every type of Ag NPs dilution. Then, in every tube of each row, we added 1, 2, or 3 ml of 1% chitosan solution. Final concentration of chitosan and Ag NPs in tested tubes is shown in Table 1.

**Table 1:** Concentrations of chitosan and Ag NPs in experimental solutions

Chitosan concentration, μg/ml	Ag concentration, μg/ml							
3.3	9.6	4.8	2.4	1.2	0.6	0.3	0.15	0.075
5.0	9.6	4.8	2.4	1.2	0.6	0.3	0.15	0.075
6.0	9.6	4.8	2.4	1.2	0.6	0.3	0.15	0.075

Test bacterial strains were grown in an appropriate broth, washed once in sterile saline, and diluted in distilled water. The bacterial concentration was standardized to an optical density of 0.08 at 600 nm (approximately 1.5 × 10^8^ UFC/mL) using the McFarland scale. Then, 100 μl of *S*. *aureus* suspension was inoculated into tubes with Ag NPs, chitosan solution, and Ag NPs-chitosan solution. Tubes containing growth medium and tested samples without inoculums were used as controls. All tubes were incubated aerobically at 37 °C for 24 h. All the measures were triplicate.

## Results

### Characterization of Ag NPs and Chitosan Used for In Situ Solution Preparation

A part of synthesized Ag NPs was modified by CTAB (Ag/CTAB NPs) (in order to improve bioactivity and stability of Ag NPs dispersions). The adsorptivity of Ag NPs towards CTAB was found to be 70.0 mg/g that corresponds to CTAB content in the sample of approximately 6.54%.

**Fig. 1:**
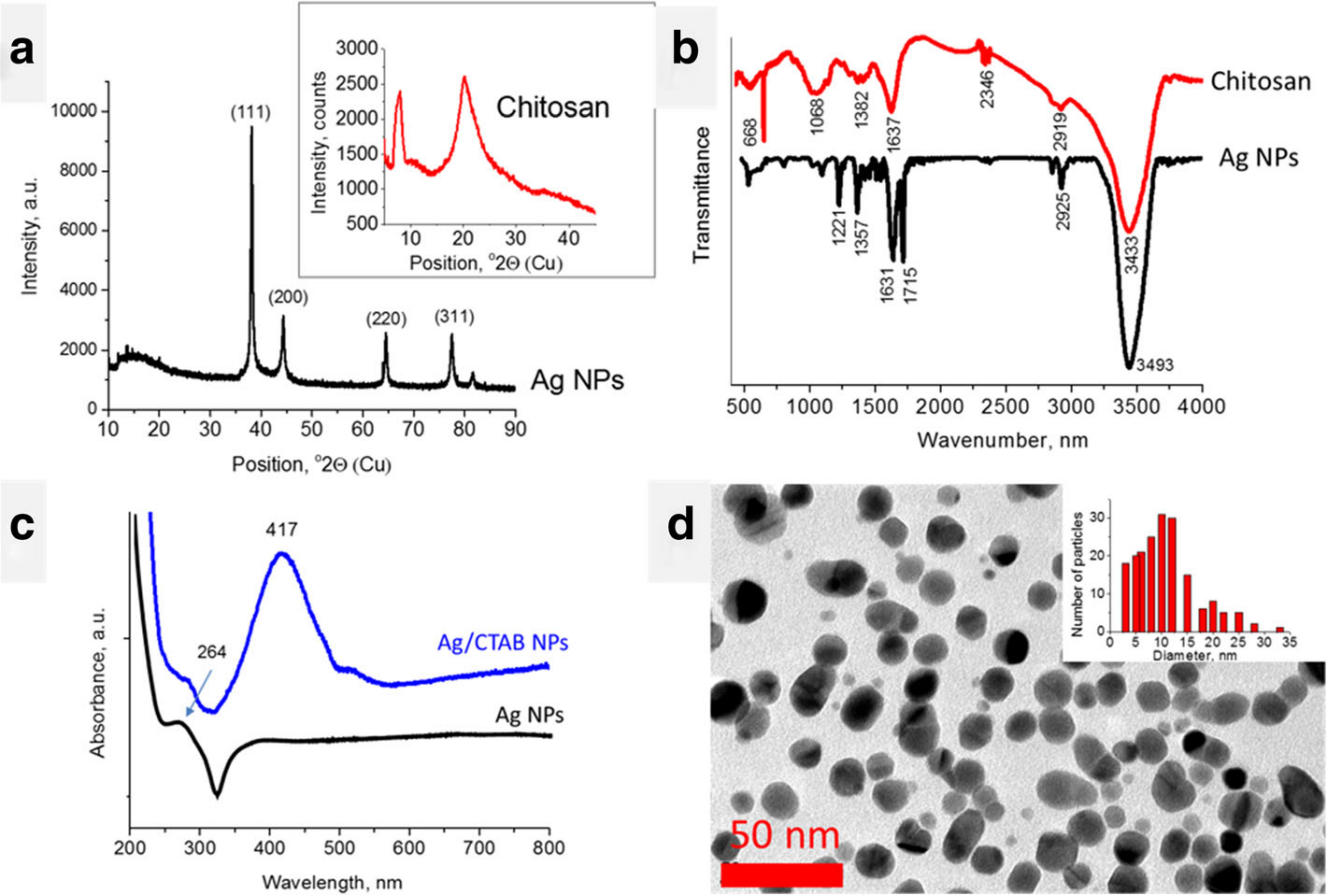
Characterization of Ag NPs and chitosan. **a** XRD patterns, **b** FTIR spectra, **c** UV–Vis absorbance spectrum of Ag NPs (water), **d** TEM image of Ag NPs

The results of XRD measurements of Ag NPs showed the presence of four sharp peaks at 38.15, 44.33, 64.48, 77.47, and 81.54 °2Theta (Fig. 1a). According to the American Mineralogist Crystal Structure Database (AMCSD) [5], these peaks were attributed to the silver. The wide peak within 12.00–21.06 °2Theta may be attributed to organic compounds which originated from synthesis (L-ascorbic acid and ginger). The XRD pattern of chitosan (Fig. 1a, inset) exhibits diffraction peaks at approximately 9 and 20 °2Theta, which are typical fingerprints of semicrystalline chitosan [5]. Crystallinity of chitosan is generated from hydrogen bonds between corresponding hydroxyl and *N*-acetyl groups. Each crystalline peak characterizes crystallographic structure, which is generated from parallel and antiparallel alignments of polymeric chains or sheets. Semicrystalline chitosan has amorphous and crystalline regions.

FTIR spectra of chitosan and Ag NPs are shown in Fig. 1b. The spectrum of chitosan shows broad and intensive bands at 3450–3200 cm^− 1^ (hydrogen-bonded OH stretching vibrations) overlapped with NH stretching bands, CH stretching band at 2783 cm^− 1^, and the band for amide I at 1652 cm^− 1^ (Fig. 1b). Bending vibrations of methylene and methyl groups are also visible at *ν* = 1375 cm^− 1^ and *ν* = 1426 cm^− 1^, respectively. Absorption in the range from 1160 to 1000 cm^− 1^ has been attributed to vibrations of CO group. The band located near *ν* = 1150 cm^− 1^ is related to asymmetric vibrations of CO in the oxygen bridge resulting from deacetylation of chitosan. The bands near 1080–1025 cm^− 1^ are attributed to *ν*_CO_ of the ring COH, COC, and CH_2_OH. The small peak at ~ 890 cm^− 1^ corresponds to wagging of the saccharide structure of chitosan [11, 13].

The FTIR spectrum of Ag NPs revealed several intensive peaks at 1226, 1366, 1636, 1714, 2851, 2924, and 3438 cm^− 1^. The latter were attributed to the H-bonded OH groups. The peaks at 1226 and 1366 cm^− 1^ are due to CO and CH bending vibrations; double peak at 1636 and 1714 cm^− 1^ point to a presence of C=C and C=O groups (stretching vibrations). The peaks at 2851 and 2924 cm^− 1^ are related to CH stretching vibrations [13]. The presence of organic groups on the Ag NPs surface are due to organic compounds used for their synthesis, L-ascorbic acid and ginger, which FTIR spectra are known [10]. If we compare the spectra of the latter with the Ag NPs one, one may notice that the double peak at 1636 and 1714 cm^− 1^ is inherent to the spectrum of L-ascorbic acid and blue-shifted. The most intensive ginger peaks situated within 1000–1200 cm^− 1^ (COC vibrations) are not intensively expressed in Ag NPs spectrum. Hence, L-ascorbic acid plays the predominant role in reduction of silver ions, transferring two electrons and transforming into dehydroascorbic acid [29]. The blue-shift of L-ascorbic acid peak position gives an evidence for the chemical bonding of this molecule on the Ag NPs surface.

The UV–Vis absorbance spectrum of the Ag NPs dispersed in water (Fig. 1c) revealed the asymmetric peak at approximately 387 nm. The peak within 387–420 nm is known as the characteristic peak for Ag NPs and is usually attributed to the surface plasmon resonance effect [30]. The asymmetry of this peak (plateau) may be ascribed to fast precipitation of Ag NPs. The peak at approximately 264 nm is also known for Ag NPs and is usually related to transition of electrons to higher energy states running in Ag NPs [38]. From the other hand, UV–Vis spectrum of L-ascorbic acid also revealed a peak at 255 nm [4]. Hence, the peak at 264 nm in Ag NPs spectrum may be considered as red-shifted peak of L-ascorbic acid confirming the presence of these chemically bonded molecules on Ag NPs surface.

It is interesting that UV–Vis spectrum of Ag/CTAB NPs (Fig. 1c, blue line) revealed a symmetric peak at 417 nm. This confirmed that the stability of Ag NPs in water was improved due to surface modification by CTAB molecules.

TEM measurements revealed that Ag NPs have roundish shape and the majority of them are 10–12 nm in size (Fig. 1d).

### Antibacterial Activities of the In Situ Prepared Chitosan/Ag NPs Solutions Against Methicillin-Resistant Strains of *Staphylococcus aureus*

MIC of pure Ag NPs and Ag/CTAB NPs against 100% MRSA was 9.6 μg/ml. The lowest concentrations have shown lesser activities (Table 2). Chitosan solution demonstrates antibacterial activities with MIC 6 μg/ml against 100% clinical strains of MRSA. Among them, 60% of strains had MIC 3.3 and 5 μg/ml chitosan solution.

**Table 2:** Antibacterial activity of pure Ag NPs to multi-resistant strains of *S*. *aureus*

The percentage of sensitive strains (%)						
Ag concentration, μg/ml	9.6	4.8	2.4	1.2	0.6	0.3
Ag NPs	100	80	60	20	20	0
AgNPs-CTAB	100	70	40	10	0	0

**Fig. 2:**
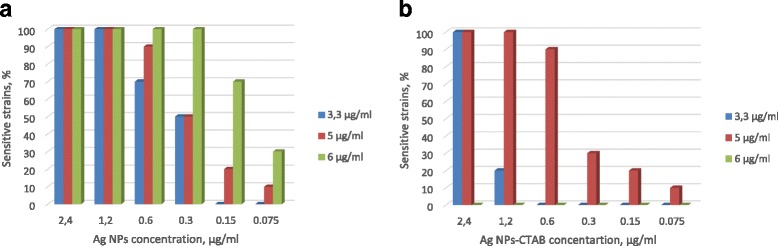
The percentage of sensitive strains of MRSA after treatment. Chitosan-Ag NPs solution (**a**) and chitosan-AgNPs-CTAB solution (**b**). 3.3, 5, and 6 μg/ml—these are the concentrations of chitosan in solution

The inhibitory effect of the chitosan-Ag NPs solution against MRSA is presented in Fig. 2a. It was found that chitosan-Ag NPs solution showed superior antimicrobial efficacy compared to its pure forms. At the same time, in situ preparation of chitosan-Ag NPs/CTAB solution (chitosan 6.0 μg/ml, Ag/CTAB NPs) was not possible due to the precipitation of the components: formation of gray-black ring agglutination and separation of the components into two phases. Antibacterial activity could not be evaluated in this case. Taking into account the unexpected result of chitosan and CTAB mixing and the lowest antibacterial activity of Ag NPs-CTAB (see Fig. 2b), we concluded that Ag NPs surface modification by CTAB is not promising. The presence of CTAB molecules on Ag NPs surface improved the stability of water dispersions, however significantly decreased antimicrobial activity and caused solution precipitation.

## Discussion

Toxicity refers to any harmful impacts on an organism during exposure to nanoparticles and their salts. If the aim is to sterilize or disinfect a specific organism, toxicity may be interpreted as a positive result (antibacterial, antiviral) [15]. The current fundamental need in nanotechnology is the development of eco-friendly and reliable methods for synthesis of metallic nanoparticles. We have affirmed the use of biological reducing agents that are natural, low-cost, and ecofriendly materials for producing silver nanoparticles, to avoid the presence of risky and toxic solvents [37]. The use of Ag NPs as therapeutic agents is limited because of their cytotoxicity against mammalian cells. Several factors can have impact on the effect of Ag NPs against microorganisms, such as size, shape, stability, and concentration of Ag NPs [4].

In our research, we obtained Ag NPs with size of 5–18 nm. It is one of the most fundamental parameters affecting the optical [39], antimicrobial [27], and antiviral properties of Ag NPs [21]. Smaller particles display greater antibacterial activity. Some studies revealed that the NPs greater than 10 nm accumulate on the cellular surface and compromise cellular permeability; however, NPs smaller than 10 nm penetrate into the bacteria, affecting DNA and the enzymes leading to cellular death [14]. It is interesting to note that although the majority of the results proved that the hypothesis of toxicity increases with decreased particle size, there are also experimental data showing that smaller NPs were either less toxic or had no size-dependent toxicity [15]. There are many studies which showed the antimicrobial activity of the Ag NPs with the range of their size from 3 to 100 nm [19].

As mentioned earlier, the effects of chitosan on the stability and antimicrobial properties of the synthesized Ag NPs were evaluated. Prior to susceptibility testing, the synthesized nanoparticles were subjected to different methods of characterization to determine their purity. Our research showed that Ag NPs in concentration of 9.6 μg/ml are effective against 100% of MRSA strains and CTAB did not increase Ag NPs effectiveness.

It is known that chitosan has significant antibacterial activity against a broad spectrum of bacteria [2]. Despite this, some reports indicate that pure chitosan does not prevent severe infections [3]. There have been several publications that have reported various combinations of chitosan and silver with improved antimicrobial properties [11]. Silver-chitosan nanocomposites were proposed as coatings for biomedical-engineering and food-packaging applications and wound-dressing applications [2, 3]. But there are limited data about the antibacterial effect of chitosan-Ag NPs solution against MRSA [34]. Our data demonstrates that simple mixing of Ag NPs in chitosan solution can enhance antibacterial activity of both components. We get increase of all investigated substance antibacterial activities. MIC of chitosan was 3.3 μg/ml and pure Ag NPs MIC and Ag NPs with CTAB MIC were 1.2 and 2.4 μg/ml, respectively. Kaur et al. (2013) also reported antibacterial activity of silver/chitosan nanocomposites against *S*. *aureus*, in which they showed similar results [36], but they did not determine the MIC. This finding demonstrates effectiveness of chitosan-Ag NPs solution, but we did not see advantages of CTAB as an antibacterial agent. On the contrary, another study showed that Ag NPs stabilized with CTAB has pronounced antibacterial effect against *S*. *aureus* and *Escherichia coli*. Probably, in our experiment, chitosan links with CTAB that decreases Ag NPs effect for bacterial cells.

## Conclusions

In this study, the activity of in situ prepared chitosan-Ag NPs solutions with different component ratios were tested against MRSA isolated from patients. Our results showed that simple mixing of the chitosan solution and Ag NPs reduces the minimal inhibition concentration of the substances into 2- and 4-folds (3.3 and 1.2 μg/ml), respectively. This result is very promising and may be considered as an effective solution in fighting against drug-resistant bacteria. It is also a progress in the direction towards personalized medicine. Future cytotoxicity study of chitosan-Ag NPs solution would give an answer about doses suitable for clinical use.

## Abbreviations

Ag NPs: Silver nanoparticles
ARI: Acute respiratory infections
CTAB: Cetrimonium bromide
FTIR: Fourier transform infrared spectroscopy
MRSA: *M*ethicillin-resistant strains of *Staphylococcus aureus*
TEM: Transmission electron microscopy
UV–Vis: Ultraviolet–visible spectroscopy
XRD: X-ray diffraction
